# Is There a Difference in Brain Functional Connectivity between Chinese Coal Mine Workers Who Have Engaged in Unsafe Behavior and Those Who Have Not?

**DOI:** 10.3390/ijerph19010509

**Published:** 2022-01-03

**Authors:** Fangyuan Tian, Hongxia Li, Shuicheng Tian, Chenning Tian, Jiang Shao

**Affiliations:** 1 Institute of Safety Management & Risk Control, Institute of Safety & Emergency Management, School of Safety Science and Engineering, Xi’an University of Science and Technology, Xi’an 710054, China; 18120089008@stu.xust.edu.cn (F.T.); tiansc@xust.edu.cn (S.T.); 19120089021@stu.xust.edu.cn (C.T.); 2 School of Management, Xi’an University of Science and Technology, Xi’an 710054, China; 3 School of Architecture & Design, China University of Mining and Technology, Xuzhou 221116, China; shaojiang@cumt.edu.cn

**Keywords:** Chinese coal mine workers, unsafe behavior, fNIRS, functional connectivity

## Abstract

(1) Background: As a world-recognized high-risk occupation, coal mine workers need various cognitive functions to process the surrounding information to cope with a large number of perceived hazards or risks. Therefore, it is necessary to explore the connection between coal mine workers’ neural activity and unsafe behavior from the perspective of cognitive neuroscience. This study explored the functional brain connectivity of coal mine workers who have engaged in unsafe behaviors (EUB) and those who have not (NUB). (2) Methods: Based on functional near-infrared spectroscopy (fNIRS), a total of 106 workers from the Hongliulin coal mine of Shaanxi North Mining Group, one of the largest modern coal mines in China, completed the test. Pearson’s Correlation Coefficient (*COR*) analysis, brain network analysis, and two-sample *t*-test were used to investigate the difference in brain functional connectivity between the two groups. (3) Results: The results showed that there were significant differences in functional brain connectivity between EUB and NUB among the frontopolar area (*p* = 0.002325), orbitofrontal area (*p* = 0.02102), and pars triangularis Broca’s area (*p* = 0.02888). Small-world properties existed in the brain networks of both groups, and the dorsolateral prefrontal cortex had significant differences in clustering coefficient (*p* = 0.0004), nodal efficiency (*p* = 0.0384), and nodal local efficiency (*p* = 0.0004). (4) Conclusions: This study is the first application of fNIRS to the field of coal mine safety. The fNIRS brain functional connectivity analysis is a feasible method to investigate the neuropsychological mechanism of unsafe behavior in coal mine workers in the view of brain science.

## 1. Introduction

A growing number of studies and investigations have shown that human unsafe behaviors and errors are the main and direct cause of accidents [1,2,3]. As early as 1931, Heinrich pointed out that 88% of accidents are attributable to unsafe human behavior [2]. In China’s coal mining industry, more than 95% of accidents are caused by the unsafe behavior of coal mine workers [4]. Thus, coal miners are considered to be one of the riskiest occupations in the world [5,6]. According to the Human Factors Analysis and Classification System (HFACS), an individual’s adverse mental state is an essential precondition for unsafe personal behavior [7,8,9]. To effectively reduce the error rate and injury rate of coal mine workers and to enhance coal mine safety management practices, it is necessary to identify and monitor the mental state of coal mine workers who have engaged in unsafe behavior. Therefore, research tools from neuroscience and cognitive psychology need to be brought into the study of coal mine workers’ unsafe behavior to further explore the neuropsychological mechanism of coal mine workers’ unsafe behavior.

The human brain is a complex network with various brain regions that process or integrate with other brain regions to perform different functions [10]. Describing how the nervous system implements and controls behavior is a central goal of modern neuroscience [11]. Psychologists have traditionally used self-report methods and performance on laboratory tasks to understand and predict human behavior. However, these indicators are only limited predictors of behavior in specific situations. In comparison, neuroimaging can be utilized as a complement to reveal the connection between neural activity and long-term, ecologically valid outcomes in laboratory environments [12,13]. In these years, resting-state functional near-infrared spectroscopy (fNIRS) is considered an emerging imaging technique and has shown valuable potential in exploring brain network architecture and the brain mechanisms underlying various cognitive functions [14]. Compared with functional magnetic resonance imaging (fMRI), fNIRS can be operated in a more economical, cost-effective, comfortable, safe, quiet, and portable way, and with high ecological validity [15,16,17,18,19,20,21,22]. In addition, fNIRS measures the concentration changes in oxygenated hemoglobin (oxy-Hb) and deoxygenated hemoglobin (deoxy-Hb) with a slightly higher temporal resolution than fMRI, which can provide more information about neurovascular changes in the brain [16]. It can be viewed as a valid and promising brain imaging approach to investigate applied societal problems, such as safety, children’s development, sport science [14,16,23,24,25,26].

In recent years, fNIRS has become a novel and advanced research tool for safety science. Current research shows a significant link between functional connectivity and behavior in safety-critical tasks [27]. In the field of safety research, scholars from driving, construction, aviation, and maritime operations have applied fNIRS to study workers’ unsafe behavior. In the study areas of driving, Tao Liu explored the potential of fNIRS as a new tool to examine driving behavior and analyzed the positive correlation between drowsiness and prefrontal activation [28,29]. Scholars mainly focus on the application of fNIRS to study fatigue driving and unsafe driving [25,30,31,32,33]. David Perpetuini applied sample entropy of the fNIRS signal to estimate the mental workload of drivers [34]. In the construction literature, Mo Hu utilized the fNIRS device to explore the construction hazard recognition [35], Yangming Shi used virtual reality experiments to assess workers’ stress status and task performance under different virtual training scenarios [36]. In the field of aviation, Frederic Dehais applied fNIRS and electroencephalography (EEG) to monitor the pilot’s cognitive fatigue [37]. Kevin J. Verdière detected pilots’ mental states in an automated versus manual landing scenario [38]. Amanda Liu designed a system based on fNIRS to assess the attention level and mental load of pilots [39]. In the field of maritime operations, Shiqi Fan found that the right lateral area of the prefrontal cortex (PFC) is sensitive to watchkeeping and decision-making during operational performance [27].

However, the existing literature indicates that in the unsafe behaviors of coal miners field, most scholars focused on accidents analysis, questionnaires, model analysis, factor analysis, and other empirical methods [40,41,42,43,44]. Furthermore, to the best of our knowledge, no scholars have applied experimental methods of brain science to study the unsafe behaviors of coal miners. Researchers now agree that PFC plays an important role in the organization, order, and timing of human behavior acts [45,46]. It is the key brain area of human cognitive functions which includes attention, working memory, and decision-making [11,47]. Decreased attention, working memory, and decision-making may cause unsafe behaviors such as operational errors [48]. Combining behavioral experiments, most of the current researches were focused on the area of fatigue [29,30,31,32,37,49,50], distraction and attention [51,52,53,54,55], brain load [56,57,58,59], sleep deprivation, and drowsiness [14,25,29,49,60,61] to explore the connection between PFC and unsafe behavior. In contrast to task-related responses, resting-state functional connectivity (RSFC) reflects the brain’s baseline, spontaneous and instinctive activity, and functional networks. RSFC measured with fNIRS has been proved to be a useful method for analyzing the mental state of affective disorders and autism spectrum disorders [62,63]. Furthermore, network science, based primarily on graph theory, is a powerful method for studying the architecture of complex networks; it has been widely used to study brain networks in various cognitive states and diseases [64,65]. Consistent with previous studies, we believe that brain connectivity is an effective method that can be used to automatically detect and classify mental fatigue [65]. From this, we hypothesized that functional brain connectivity is available to investigate the neuropsychological mechanism of coal mine workers’ unsafe behavior.

Thus, to fully understand the neuropsychological mechanism of coal mine workers’ unsafe behavior, it is necessary to explore the connection between coal mine workers’ neural activity and unsafe behavior. In this study, we conducted a resting-state functional near-infrared spectroscopy (rs-fNIRS) study with 106 coal mine workers in China and analyzed the functional connectivity between coal mine workers who have engaged in unsafe behaviors and those who have not, providing a new approach to analyze unsafe behaviors of coal miners and further promote the cross-fertilization of brain science and coal mine safety science.

## 2. Materials and Methods

### 2.1. Demographic Information of the Subjects

In this study, based on a random sampling method, 120 male miners from Shaanxi Coal Group Northern Shaanxi Mining Hongliulin Company, one of the largest modern coal companies in China, were selected as subjects. 14 participants were excluded because of large motion artifacts in the signals due to head movements or extreme fatigue (10 from NUB and 4 from EUB). Therefore, the effective subjects of this study were 106. Among them, 80 miners had no ‘‘three disobeying’’ behavior (NUB), the other 26 miners have engaged in ‘‘three disobeying’’ behavior (EUB) in the last three years. China’s “Special Provisions of the State Council on the Prevention of Production Safety Accidents in Coal Mines” clearly instructs that the “three disobeying” in coal mines refer to the phenomenon or behaviors of coal mine workers who: a) disobeyed the rules and regulations; b) disobeyed operation disciplines; c) disobeyed labor disciplines in the process of production and construction. Eliminating “three disobeying” behaviors is one of the most important guarantees in coal mine safety. According to the current requirements of China’s coal mines regulation, the “ three disobeying “ are generally considered to be unsafe behaviors, such as underground smoking, sleeping, going down the mine late, going up the mine early, taking off work, fatigue work, bad mood work [66,67]. Regarding the basic information of the subjects, the average age of the subjects was 27.3 ± 5.7 years, the average height was 172.00 ± 4.62 cm and the average weight was 68.14 ± 7.80 kg. Detailed information was reviewed before the study, and participants were required to have received no psychotropic medication (such as stimulants, antidepressants, and antianxiety drugs) and to have no history of neurological damage or illness, epilepsy, or psychiatric disorders. Based on the handedness scale, we confirmed that all subjects were right-handed and had a normal or correctional vision. Subjects were forbidden to drink sensitive products (such as alcohol or caffeine) 24 h before the experiment. The participants reported their body information and how much sleep they had had the night before, ensuring that they had enough sleep. During fNIRS data collection, the participants were instructed to remain still and stare at the cross directly in the center of the screen with their eyes open without falling asleep. The time duration for resting-state fNIRS data acquisition was approximately 5 min for each subject. Experimental room conditions (light and temperature) were kept constant throughout the experiment. To avoid interfering with the coal mine workers’ normal work and reduce the effect of time on the data, this study chose coal mine workers on leave to complete the experiment during 10:00–14:00. The demographic information of the subjects showed in Table 1 and Table 2.

Before the experiment, subjects were required to fully understand the contents of the experimental program, and all participants provided written informed consent. The experimental procedure was approved by the Human Ethics Committee of Xi’an University of Science and Technology and met the ethical standards stipulated in the 1975 Helsinki Declaration.

### 2.2. Data Acquisition

**Figure 1:**
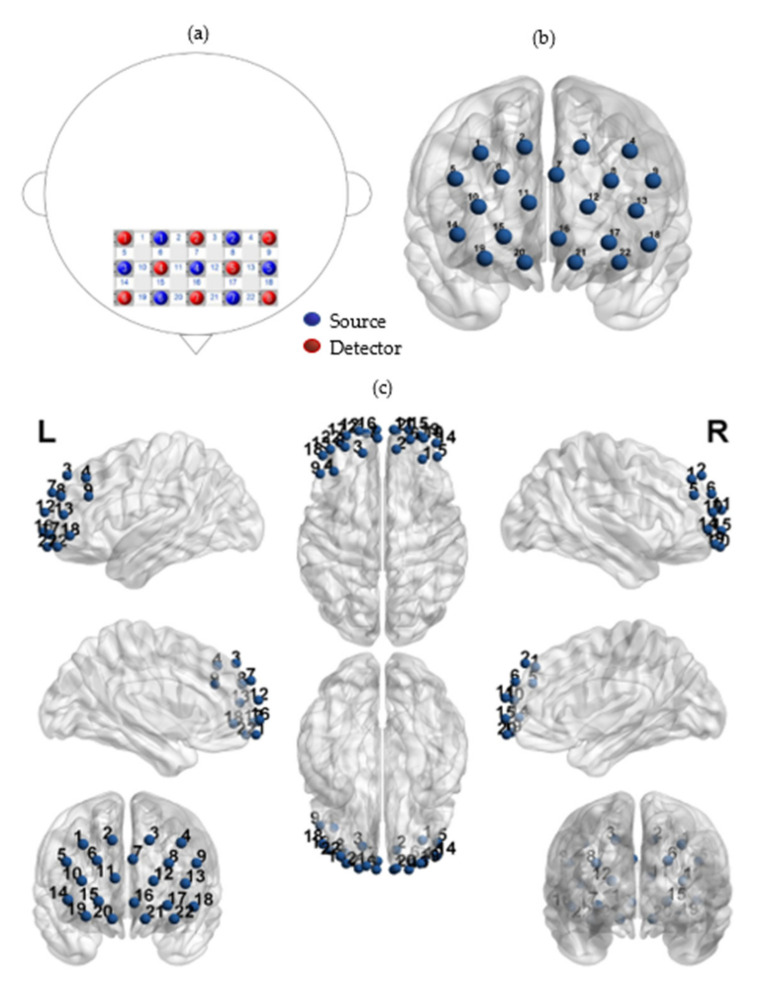
(**a**) Positions of fNIRS channels. (**b**) Fifteen optodes (eight sources and seven detectors) were attached to the forehead in a 5 × 3 lattice pattern forming 22 measurement channels (in frontal view). (**c**) The 3D MNI coordinates of the fifteen optodes in a different view.

A 22-channel continuous-wave near-infrared optical imaging system (LABNIRS; Shimadzu Corporation, Kyoto, Japan) with each source including two wavelengths (690 and 830 nm) of near-infrared light was used to measure the time course of oxy-hemoglobin (oxy-Hb) and deoxy-hemoglobin (deoxy-Hb) concentrations at a sampling rate of 7.4074 Hz [68]. The time duration for resting-state fNIRS data acquisition was approximately 5 min for each subject, including 2.224 sample points. The 8 sources and 7 detector probes were plugged into a holder and arranged into a 5 × 3 array resulting in 22 measurement channels covering the prefrontal areas (inter-optode distance = 30 mm, Figure 1). This probe design was also the same as that in the previous series of fNIRS studies [23,69,70]. Detector 7 was perpendicular to the tip of the nose and flush with the eyebrow. Measurement patches were attached to a regular swimming cap worn by the participant. Since the prefrontal cortex of the brain plays a central role in response execution, memory extraction, and emotional assessment, and is associated with socialization, perception, attention, and decision-making [11,47]. This arrangement allowed us to assess the surface portions of our main regions of interest (ROIs), PFC, including the middle parts of the dorsolateral prefrontal cortex (dlPFC) (CH01, CH02, CH03, CH04, CH05, CH08, CH13, CH14, and CH18), the frontopolar area (CH06, CH07, CH10, CH11, CH12, CH15, and CH16).

The positions of all fNIRS channels were measured by a 3D electromagnetic tracking device (FASTRAK; Polhemus, USA) after the experiment. The origin of this system is in the center of the chin. Four reference points are obtained at the nasion (Nz), right preauricular points (AR), left preauricular points (AL), and central zero (Cz) [71]. The positions of the fNIRS sources and detectors were obtained according to the origin and the four reference points. The Montreal Neurological Institute (MNI) of an fNIRS channel was computed from the positions of the sources and detectors by the MATLAB toolbox NIRS-SPM (The MathWorks Inc., Natick, MA, USA) [61,72,73]. The probability is to describe how the estimated MNI coordinates accurately correspond to the specific brain regions. The estimated mean locations of the fNIRS channels were obtained using anatomical information based on Brodmann areas. These are reported in Table 3.

### 2.3. Data Preprocessing

The fNIRS data were preprocessed using MATLAB R2013b by our script. We manually converted the a.txt file, the output from the LABNIRS system, to the a.mat file. The modified Beer-Lambert law (MBLL) was applied to compute concentration changes in hemoglobin signals from the attenuation of light through the head at two wavelengths [74]. According to Duncan’s study of 100 adults, the mean value of the differential pathlength factor (DPF) was 6.53 ± 0.99 [75]. Discrete wavelet transformation was adopted to reduce head movements and surface noise [76]. Similar to previous studies, band-pass filtering with cutoff frequencies of 0.02 and 0.08 Hz was applied to remove the long-term trends, respiratory and cardiac noises [49,70,77,78]. Compared with deoxy-Hb signals and total-Hb signals, oxy-Hb signals are a more sensitive indicator of changes associated with regional cerebral blood flow. Thus, oxy-Hb signals were selected as the research objects of this study [79,80,81].

### 2.4. Resting-State Functional Connectivity Analysis

#### 2.4.1. Pearson’s Correlation Coefficient and *t*-Test

Common analysis indicators of RSFC include Pearson’s Correlation Coefficient (*COR*), Magnitude Squared Coherence (*COH*), and Phase Locking Value (*PLV*). Among them, *COR* is the most commonly used indicator [36,77,82]. In this study, *COR* is used to describe the linear correlation between the time domain signals *x(t)* and *y(t)* of two channels. It is generally assumed that two signals have no delay. For signals with a mean of 0 and a variance of 1, *COR* can be defined as:

(1) $$COR_{xy}=\frac{1}{N}\overset{N}{\underset{k=1}{\sum }}x\left(k\right)y\left(k\right)$$

*COR* value range: [−1,1]. If the two signals are completely negative (linearly) correlated, the value is −1; if the two signals are completely positive (linear) correlated, the value is 1; if there is no linear correlation between the two signals (there may be nonlinear correlation), the value is 0.

The connection strength of the prefrontal cortex neuron population in 5 min of this experiment can be obtained, by calculating the *COR* matrix of between 22 channels of two groups [73]. The rows and columns of these 22 × 22 matrices represent the channel numbers, while the elements of the matrices were the correlation coefficients of the matching channels.

After the construction of *COR* matrix, to further explain the difference between the two groups of functional connectivity, a binary transformation of the *COR* matrix was used. Referring to previous studies, the threshold was set as 0.5 and 0.7, and *COR* greater than the threshold was defined as “1”, while *COR* less than the threshold was defined as “0” [73,83,84,85].

The *COR* matrix of 22 channels between the two groups was carefully examined using two-tailed paired *t*-tests. Multiple comparison correction was adopted to control for the probability of false-positive events, and false discovery rate (FDR) correction was performed for all *COR* results (*q* < 0.05) [86]. All statistical analyses were performed by SPSS 26.0 (SPSS Inc., Chicago, IL, USA), and the significance level was set to *p* < 0.05.

#### 2.4.2. Brain Network Analysis

Graph-theoretical topology analysis is rich in content and widely used. Many disciplines, such as communication, computer science, and neuroimaging, utilized graph theory as a tool to solve practical and theoretical problems [87]. In this study, graph theory analysis was conducted to further evaluate the functional connectivity of these 22 channels [23,69,70,73]. For complex brain networks, clustering coefficient, global efficiency, local efficiency, and small-world network measure, are often used in network topology characteristic analyses [65,80,84]. All these indicators were calculated by GRETNA on MATLAB [88]. Referring to previous studies, a widely used sparsity threshold were adopted [23,69,70,80,83,89,90]. A range of continuous threshold values (sparsity) *T* ($T\in \left(0.1:0.1:0.9\right)$), were input to construct the brain networks. Brain networks are typically compared with random networks to test whether they are configured with significantly non-random topology [88]. Further, 100 matched random networks were generated to compute the ratios of all these indicators between the real brain functional networks [84,91,92,93].

The nodes and edges are two essential components to construct the brain networks [94,95]. In this study, *N* is the set of all nodes in the network, and *n* is the number of nodes. *L* is the set of all links in the network, and *l* is the number of links. *(i, j)* is a link between nodes *i* and *j*, (*i, j∈N*). *a_ij_* is the connection status between *i* and *j*: *a_ij_=1* when link *(i, j)* exists (when *i* and *j* are neighbors); *a_ij_* = 0 otherwise (*a_ii_ =* 0 for all *i*).

The number of links were computed as $l=\underset{i,j\in N}{\sum }a_{ij}$ (to avoid ambiguity with directed links we count each undirected link twice, as *a_ij_* and as *a_ji_*) [94].

The degree is defined as the number of links connected to a node, degree of a node *i* [94]:

(2) $$k_{i}=\underset{j\in N}{\sum }a_{ij}$$

The number of triangles around a node *i*,

(3) $$t_{i}=\frac{1}{2}\underset{j,h\in N}{\sum }a_{ij}a_{ih}a_{jh}$$

Thus, the clustering coefficient is defined as follows [87,94,96]:

(4) $$C_{net}=\frac{1}{n}\underset{i\in N}{\sum }C_{i}=\frac{1}{n}\underset{i\in N}{\sum }\frac{2t_{i}}{k_{i}\left(k_{i}-1\right)}$$

where $C_{i}\;$is the clustering coefficient of node *i* ($C_{i}$ = 0 for *k_i_ < 2*). The clustering coefficient evaluates the local clustering or region range of the network. A network with a larger clustering coefficient indicates a more isolated network topology [65].

The shortest path length (distance), between nodes *i* and *j*:

(5) $$d_{ij}=\underset{a_{uv}\in gi↔j}{\sum }a_{uv}$$

where *g_i↔j_* is the shortest path (geodesic) between *i* and *j*. Note that $d_{ij}=\infty \;$for all disconnected pairs *i, j*.

The characteristic path length of network [84]:

(6) $$L_{p}=\underset{i\in G}{\sum }\frac{\sum_{i\neq j\in G}min\left\{L_{i,j}\right\}}{\left(N-1\right)N}$$

where $min\left\{L_{i,j}\right\}\;$is the shortest path (geodesic) between node *i* and node *j*, *G* is the set of all nodes in the network. The characteristic path length is the average of the shortest path lengths between any pair of areas in the network; it measures the overall route effectiveness of the network. Networks with short characteristic path lengths indicate a high efficiency of parallel information transmission [65].

The small-world network measure [84,87,94]:

(7) $$\sigma =\frac{\gamma }{\lambda }$$

where $\gamma =C_{net}/C_{random}\;,$ $\lambda =L_{net}/L_{random}$, *C* and $C_{random}\;$are the clustering coefficients, *AL* and $AL_{random}$ are the characteristic path lengths of the respective tested network and a random network. $C_{random}$ and $L_{random}$ denotes the average clustering coefficient and characteristic path length of 100 matched random networks, respectively, which possess the same number of nodes, edges, and degree distribution as the real brain network [97,98]. Small-world networks often have σ≫1.

The global efficiency [94,99]:

(8) $$E_{global}=\frac{1}{N\left(N-1\right)}\underset{i,j,i\neq j}{\sum }\frac{1}{d_{ij}}$$

where $d_{ij}\;$is the shortest path length (distance), between nodes *i* and *j*.

The local efficiency [94,99]:

(9) $$E_{loc}=\frac{1}{n}\underset{i\in N}{\sum }E_{loc,i}=\frac{1}{n}\underset{i\in N}{\sum }\frac{\sum_{j,h=N,j\neq i}a_{ij}a_{ih}{\left[d_{jh}\left(N_{i}\right)\right]}^{-1}}{k_{i}\left(k_{i}-1\right)}$$

where $E_{loc,i}$ is the local efficiency of node *i*, and $d_{ih}\left(N_{i}\right)$ is the length of the shortest path between *j* and *h*, that contains only neighbors of *i*.

The Two-Sample T-test was utilized to confirm the differences between safe workers and unsafe workers in $C_{net}$, $S_{net}$ and $E_{global}$. The significance of the data was tested with a confidence level of *p* < 0.05.

## 3. Results

### 3.1. Demographic Information

Table 1 illustrates the demographic information for NUB and EUB. Overall, the mean length of service was longer in EUB (9.76 ± 7.02) than in NUB (9.00 ± 7.06); the mean height was essentially the same in NUB (172.88 ± 5.01) and EUB (171.71 ± 4.49); the mean age was greater in EUB (36.38 ± 6.40) than in NUB (34.89 ± 6.77), and the mean weight was higher in NUB (69.50 ± 7.04) was higher than that of EUB (67.73 ± 7.97). The marital status and education information of NUB and EUB was shown in Table 2. In terms of distribution rate, the proportion of unmarried is higher in EUB (19.2%) than in NUB (10.0%). Compared to NUB, there are more coal mine workers with low educational attainment in EUB, accounting for 34.6%.

Unfortunately, the results of the chi-square test showed that there was no significant difference between the two groups of coal mine workers in the length of service, height, age, weight, marital status, and education information. One-way ANOVA results showed no significant differences in coal mine workers’ brain functional connectivity among the above demographic factor subgroups.

### 3.2. Pearson’s Correlation Coefficient and t-Test

**Figure 2:**
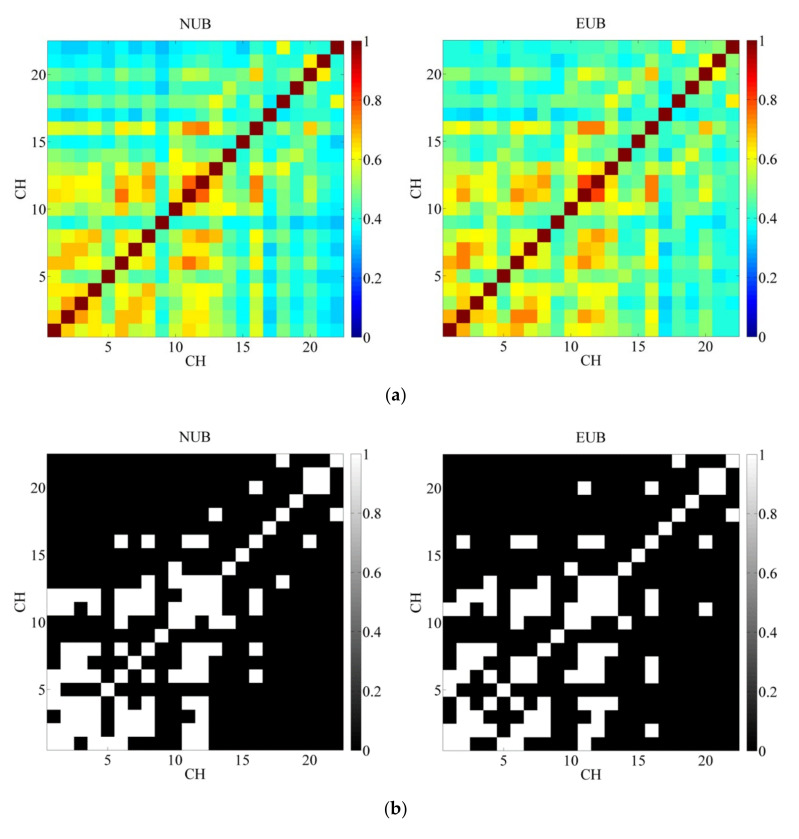

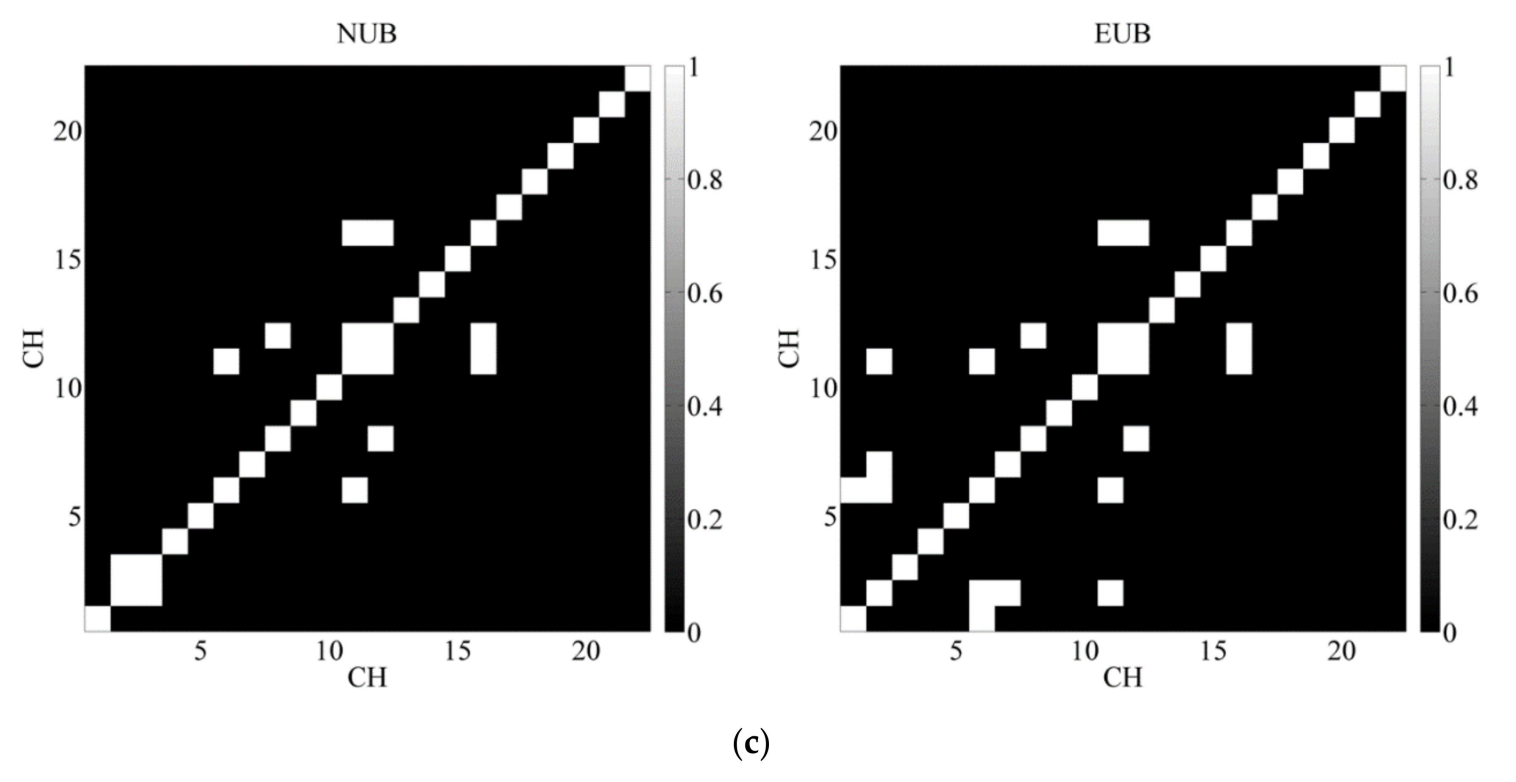
(**a**) Functional connectivity matrices between any two channels for NUB and EUB. (**b**) Binary matrices with *COR* = 0.5. (**c**) Binary matrices with *COR* = 0.7.

Figure 2a shows the 22 × 22 correlation matrices for NUB and EUB. Each grid represents the functional connectivity (*COR*∈ [0, 1]) between the two channels. A larger *COR* indicates a stronger correlation between channels, meaning that activation of one channel is significantly correlated with activation of the other. In Figure 2a, blue indicates weak connections between channels, and red indicates strong connections. In Figure 2b,c, based on the binary method, *COR* = 0.5 and *COR* = 0.7 were, respectively, set as critical values in this study. If the *COR* between two channels was less than the critical value, it was black; if it was greater than the critical value, it was white. It can be seen that stronger connectivity EUB was shown in comparison to NUB (*p* < 0.05 two-sampled *t*-test).

In particular, Figure 2b described the binary matrices (*COR* = 0.5) of NUB and EUB, the functional connectivity of NUB was 42.98% while that of EUB was 43.39%. In these two groups, the channels with *COR* greater than 0.5 were mainly concentrated in Brodmann’s Areas (BA) 9 (CH01, CH02, CH03, CH04), BA 10 (CH06, CH07, CH10, CH11, CH12, CH16), and BA 46 (CH08, CH13, CH14). That is, the strength of the functional connection between the dorsolateral prefrontal cortex (BA 9 and BA 46) and frontal pole area (BA 10) was stronger than other brain areas in PFC.

The functional connectivity of NUB in Figure 2c was 7.02% area wise while that of EUB was 8.26% (*COR* = 0.7). In this case, the differences between the two groups were significantly concentrated in CH 02-03 (BA 9), CH 06-01 (BA 10-BA 9), CH 06-02 (BA 10-BA 9), CH 07-02 (BA 10-BA 9) and CH 11-02(BA 10-BA 9). In other words, the *COR* in the frontal pole area and dorsolateral prefrontal cortex of EUB were stronger than that of NUB.

**Figure 3:**
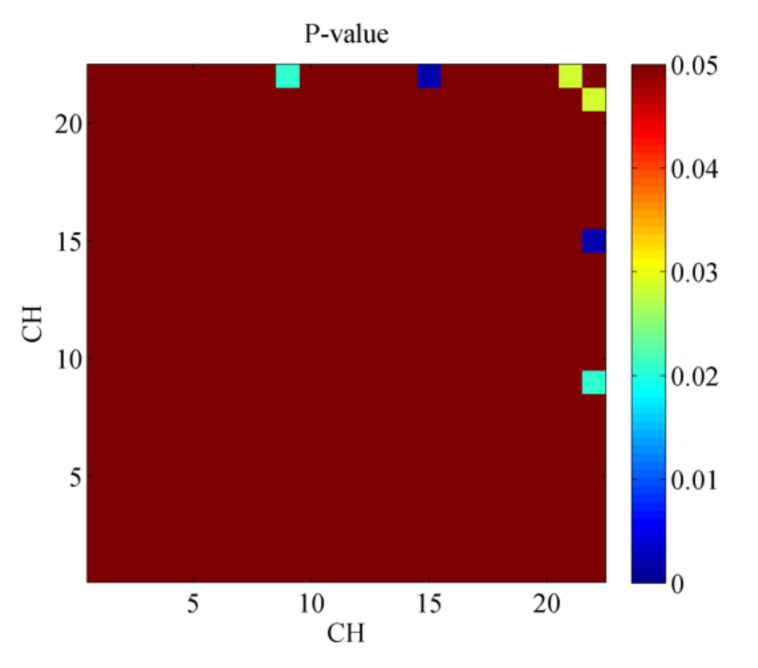
*p*-value of functional connectivity matrices for NUB and EUB (*p* < 0.05).

As shown in Figure 3, it is clear that among the functional connectivity matrices for NUB and EUB there were three pair channels passed the two-sample test (*p* < 0.05). Specifically, CH 15-22 (*p* = 0.002325), CH 09-22 (*p* = 0.02102), and CH 21-22 (*p* = 0.02888). In this study, CH 9 belongs to BA 45, CH 15 belongs to BA 10, CH 21, and CH 22 belongs to BA 11. In other words, the frontopolar area and orbitofrontal area (CH 15-22), pars triangularis Broca’s area and orbitofrontal area (CH 09-22), Orbitofrontal area (CH 21-22) were the regions with the difference between the functional connection matrix of NUB and EUB.

**Figure 4:**
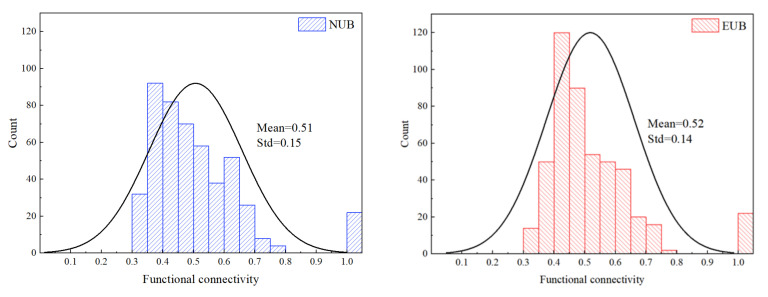
Histograms of the functional connectivity distribution of NUB and EUB.

Figure 4 shows the histograms of the functional connectivity distribution of NUB and EUB. The mean and standard deviation (Std) of functional connectivity of the two groups were similar, while the frequency distribution is quite different, especially in the range 0.35 to 0.5.

### 3.3. Brain Network Analysis

**Figure 5:**
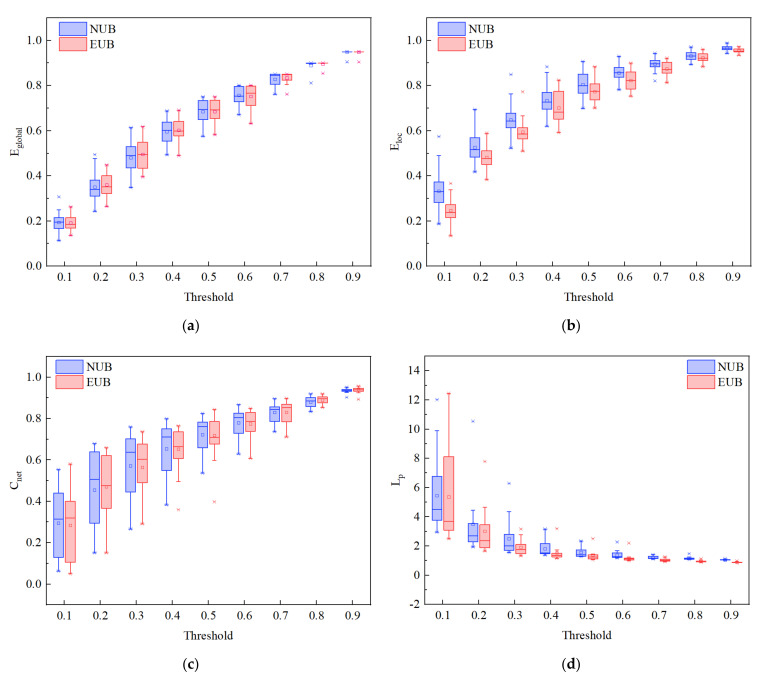
Comparison of the NUB brain network to the EUB brain network: network efficiency. (**a**) The global efficiency ($E_{global}$). (**b**) The local efficiency ($E_{loc}$). (**c**) The clustering coefficient ($C_{net}$). (**d**) The characteristic path length ($L_{p}$). The blue color represents NUB anticipation, and the red color represents EUB anticipation. The horizontal axes show the threshold values (*T*∈(0.1:0.1:0.9)) and the vertical axes show the network properties indexes (*p* < 0.05).

For the subsequent network analysis, the correlation matrices were thresholded over a sparsity range from 0.1 to 0.9. As a function of network efficiency, the global efficiency ($E_{global}$), the local efficiency ($E_{loc}$), the clustering coefficient ($C_{net}$), and the characteristic path length ($L_{p}$) are depicted in Figure 5. In general, the parameters of global efficiency (Figure 5a) and local efficiency (Figure 5b) increased with threshold, which is consistent with the previous findings [93,100]. For brain networks of NUB and EUB, the clustering coefficients increased (Figure 5c), but the characteristic path length decreased (Figure 5d) as sparsity increased. Consistent with previous studies, these results indicate that the PFC functional network has stable small-world characteristics [65,89,93,100,101].

The oxy-Hb-based group differences in clustering coefficient, nodal efficiency, and nodal local efficiency during resting state were available in Table 4, provided with a two-sample test (*p* < 0.05). It is worth noting that among these three network metrics, only CH 08 (belongs to BA 46) passed the two-sample *t*-test, specifically, the clustering coefficient (*p* = 0.0004), nodal efficiency (*p* = 0.0384), nodal local efficiency (*p* = 0.0004).

**Figure 6:**
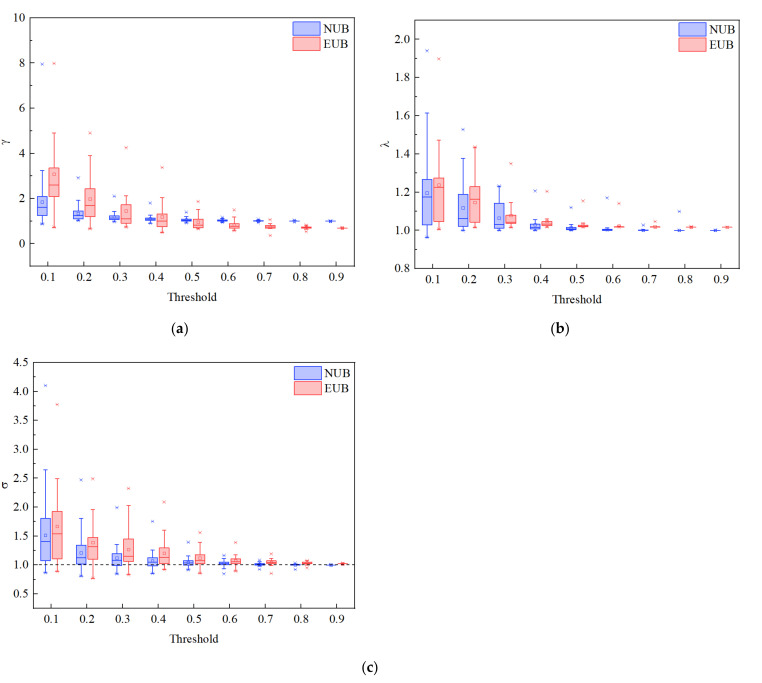
Comparison of the NUB brain network to the EUB brain network: small-worldness. Brain network data were based on the functional networks generated from the oxy-Hb measurements over continuous threshold values ((T∈(0.1:0.1:0.9)). (**a**) γ. (**b**) λ. (**c**) σ.

The small-world analysis results were displayed in Figure 6, in which we discovered that the γ (Figure 6a), λ (Figure 6b) and σ (Figure 6c), respectively, decreased and descended with increased sparsity threshold for both NUB and EUB networks. Further, since the λ was larger than 1 and the γ approached to 1 (σ>1), both the resting-state brain networks in NUB and EUB exhibited the small-world properties.

## 4. Discussion

In this study, a resting-state fNIRS measurement was utilized to characterize and identify the differences in functional connectivity and brain network indicators in the PFC regions between NUB and EUB. To the best of our knowledge, this study is the first to use fNIRS in the coal mine safety field. Overall, our findings demonstrated that the fNIRS brain functional connectivity and brain network analysis is a new approach that could be used to further explore the neuropsychological mechanism of coal mine workers’ unsafe behavior from the perspective of brain science. For each subject, we recorded 5 min of continuous fNIRS data of PFC and applied band-pass filtering to eliminate physiological noise. First, we applied *COR* to discriminate the difference in brain functional connectivity between NUB and EUB. In general, EUB has higher connectivity on PFC than NUB, especially in the frontal pole area and dorsolateral prefrontal cortex. Further, the difference between NUB and EUB 22 × 22 channels’ *COR* was examined using a two-tailed *t*-test (*p* < 0.05). The results showed a significant difference in *COR* between NUB and EUB in the frontopolar area and orbitofrontal area (CH 15–22), pars triangularis Broca’s area, and orbitofrontal area (CH 09–22), Orbitofrontal area (CH 21–22). In addition, we also discovered that both NUB and EUB cases exhibited small-world properties. More importantly, only CH 08 (belongs to the dorsolateral prefrontal cortex) passed the two-sample *t*-test among the 22 channels, which indicated that the brain networks of NUB and EUB show significant differences in CH 08.

With respect to the connectivity patterns of NUB and EUB, our analysis demonstrated that the resting-state functional connectivity of EUB in the frontal pole area and dorsolateral prefrontal cortex were connected more intensively in the ROIs of PFC. It has been proved that executive function and selective attention are related to the dorsolateral prefrontal cortex, and multitasking ability is related to the frontopolar area [102,103,104,105]. Therefore, it can be inferred that the executive function, selective attention, and multitasking ability of EUB are more closely related than that of NUB. Previous studies have shown that with increasing cognitive load, hemodynamic activity levels increase in the PFC with enhanced brain functional connectivity (*COR*) [33]. In addition, higher severity of depression was related to increased dynamic RSFC in the dorsolateral prefrontal cortex [106]. Impulsive traits are linked to increased functional connectivity within the dorsolateral prefrontal cortex [107]. These pieces of evidence are consistent with our research, coal mine workers with depression, impulsion, or had a cognitive overload were usually easier to engage in unsafe behavior. Interestingly, Guillermo Borragán explores the reduction in functional connectivity between left prefrontal cortical regions caused by a sustained attentional decline after cognitive fatigue induction in the presence of high sleep pressure [49]. A possible reason for the inconsistency between our results and previous results is that Guillermo Borragán’s experiment was under special conditions (after the whole night sleep deprivation) while our experiment was conducted in the noon with enough sleep.

In a comparison between the two resting states, two-sample *t*-test at 95% confidence interval showed that CH 15–22 (*p* = 0.002325), CH 09–22 (*p* = 0.02102), and CH 21–22 (*p* = 0.02888). In other words, there are significant differences between NUB and EUB in the brain functional connections between the three groups of channels. Specifically, CH 15–22 represents the frontopolar area and orbitofrontal area, CH 09–22 represents pars triangularis Broca’s area and orbitofrontal area, and CH 21–22 represents the orbitofrontal area. The dorsolateral prefrontal cortex, frontopolar area, and orbitofrontal area work together for central executive functions [108]. It is also responsible for systems, such as response execution, memory extraction, and emotional assessment, and is associated with socialization, perception, attention, and decision-making [11,47]. Broca’s area is related to semantic judgment [109]. From this, it is reasonable to infer that NUB and EUB differ in response execution, memory extraction, and emotion, socialization, perception, attention, decision making, and semantic judgment. Combined with the field interviews by our research team in Shaanxi Coal Group Northern Shaanxi Mining Hongliulin Company, we learned that those coal mine workers who engaged in unsafe behavior mostly have poor concentration, bad reaction and execution skills, and emotional instability. It follows that our experimental results are consistent with the behavioral performance of coal mine workers.

On this basis, we analyzed the brain network differences between NUB and EUB. The results showed that the trends of parameters such as global efficiency, local efficiency, clustering coefficients, and feature path length of the two groups under different thresholds were consistent with previous studies, but no significant differences were found [65,89,93,100]. Further, we calculated the small-world properties of the brain networks of NUB and EUB separately, and the results were greater than 1. That is, the resting-state brain network data of both groups have small-world properties. Notably, the results of the two-sample *t*-test showed that only CH 08 (dorsolateral prefrontal cortex) passed the significance test for both clustering coefficient, nodal efficiency, and nodal local efficiency (*p* < 0.05). Mengjing Wang has proved that the clustering coefficient was of excellent level reliability [70]. Previous studies showed that the dorsolateral prefrontal cortex is considered to be related to attention control during executive functions [110]. The higher clustering coefficient represents increased connectivity strength among neighbor nodes and a higher processing rate of local information [111]. Previous evidence showed that higher brain network clustering is associated with superior working memory [112]. Thus, it is possible to reasonably infer that compared to EUB, NUB has stronger attention control and better working memory to ensure safe work. In the following works, further experimental tests could be performed on NUB and EUB in the dorsolateral prefrontal cortex to examine the stability of this region in distinguishing the significance of NUB and EUB.

Several limitations of the present work must be addressed. First, this study was undertaken in a well-controlled laboratory environment. In the real world, underground coal mine shafts and coal mine work operations are more complex and unpredictable. Therefore, more detailed and dynamic scenarios need to be measured in future studies. Second, the limited sample size of this study may affect the accuracy of the analysis results. Third, this study concentrated only on the PFC, which could ignore the influences of other brain regions. In fact, the frequency of unsafe behavior varies in coal mine workers on different shifts, our next study will further explore the mechanisms underlying the occurrence of unsafe behavior in coal mine workers by shift.

## 5. Conclusions

This rs-fNIRS study confirmed that the differences in brain functional connectivity between coal mine workers who have engaged in unsafe behaviors and those who have not. On the one hand, the *COR* analysis of NUB and EUB were significantly different among the frontopolar area, orbitofrontal area, and pars triangularis Broca’s area. On the other hand, brain network analysis results showed significant differences in clustering coefficient, nodal efficiency, and nodal local efficiency in the dorsolateral prefrontal cortex. Altogether, the results showed that the fNIRS functional connectivity is feasible to investigate the neuropsychological mechanism of unsafe behavior of coal mine workers. Future research can introduce other metrics describing the temporal time course of the hemoglobin variations and machine learning approaches to further explore the neuropsychological mechanism of unsafe behavior of coal mine workers.

## Figures and Tables

**Table 1:** The demographic information of 106 subjects, Chi-square test, and one-way ANOVA test.

	NUB (*n* = 80)	EUB (*n* = 26)	Chi-Square Test		One-Way ANOVA	
	Mean ± Std	Mean ± Std	χ^2^	*P*_1_	*P*_2_	*F*
Length of service/year	9.00 ± 7.06	9.76 ± 7.02	0.831	1.000	0.961	0.154
Height/cm	172.88 ± 5.01	171.71 ± 4.49	1.319	0.966	0.304	1.226
Age/year	34.89 ± 6.77	36.38 ± 6.40	1.855	0.562	0.540	0.724
Weight/kg	69.50 ± 7.04	67.73 ± 7.97	1.306	0.802	0.199	1.579
Marital status	-	-	0.283	0.868	0.812	0.209
Education information	-	-	1.442	0.780	0.831	0.368

Note: *P*_1_ < 0.05, samples passed the Chi-square test. *P*_2_ < 0.05, samples passed the one-way ANOVA test.

**Table 2:** The marital status and education information of 106 subjects.

	NUB (*n* = 80)		UW (*n* = 26)	
	*n*	%	*n*	%
Marital status				
Divorced	1	1.2	0	0
Married	71	88.8	21	80.8
Unmarried	8	10.0	5	19.2
Education information				
Bachelor’s degree	6	7.5	3	11.5
College	12	15.0	5	19.2
High school	39	48.8	9	34.6
Junior high school	1	1.3	0	0
Technical secondary school	22	27.5	9	34.6

**Table 3:** Locations of all fNIRS channels.

CH	Brodmann Area	MNI Coordinates			Probability
		x	y	z	
**CH01**	* 9—Dorsolateral prefrontal cortex	31	48	42	0.7547
**CH02**	* 9—Dorsolateral prefrontal cortex	11	58	41	0.9958
**CH03**	* 9—Dorsolateral prefrontal cortex	−12	58	41	1.0000
**CH04**	* 9—Dorsolateral prefrontal cortex	−29	48	39	0.6406
**CH05**	* 46—Dorsolateral prefrontal cortex	43	51	28	0.6368
**CH06**	* 10—Frontopolar area	21	65	28	0.6324
**CH07**	* 10—Frontopolar area	−1	64	26	0.8878
**CH08**	* 46—Dorsolateral prefrontal cortex	−22	63	28	0.8619
**CH09**	45—pars triangularis Broca’s area	−39	51	27	0.9889
**CH10**	* 10—Frontopolar area	31	67	14	0.8810
**CH11**	* 10—Frontopolar area	12	72	16	1.0000
**CH12**	* 10—Frontopolar area	−13	72	15	1.0000
**CH13**	* 46—Dorsolateral prefrontal cortex	−31	64	16	0.7677
**CH14**	* 46—Dorsolateral prefrontal cortex	40	64	0	0.3726
**CH15**	* 10—Frontopolar area	21	72	3	0.4762
**CH16**	* 10—Frontopolar area	−4	71	4	0.7357
**CH17**	* 11—Orbitofrontal area	−21	72	5	0.6826
**CH18**	* 46—Dorsolateral prefrontal cortex	−39	62	3	0.9173
**CH19**	* 11—Orbitofrontal area	29	69	−8	0.8404
**CH20**	* 11—Orbitofrontal area	11	73	−7	0.9443
**CH21**	* 11—Orbitofrontal area	−13	72	−6	0.9964
**CH22**	* 11—Orbitofrontal area	−32	66	−6	0.5135

Note: * indicates Region of Interest.

**Table 4:** Group differences in clustering coefficient, nodal efficiency and nodal local efficiency during resting state.

		Clustering Coefficient				Nodal Efficiency				Nodal Local Efficiency			
ROI	CH	Mean ± Sd		T-Value	*p*-Value	Mean ± Sd		T-Value	*p*-Value	Mean ± Sd		T-Value	*p*-Value
		NUB	EUB			NUB	EUB			NUB	EUB		
* 9—Dorsolateral prefrontal cortex	01	0.6 ± 0.1	0.59 ± 0.11	0.4812	0.6314	0.56 ± 0.01	0.57 ± 0.09	−0.3753	0.7082	0.66 ± 0.11	0.65 ± 0.12	0.3351	0.7382
* 9—Dorsolateral prefrontal cortex	02	0.64 ± 0.1	0.62 ± 0.08	0.8237	0.4120	0.56 ± 0.01	0.59 ± 0.07	−1.7021	0.0917	0.69 ± 0.09	0.69 ± 0.06	0.0159	0.9873
* 9—Dorsolateral prefrontal cortex	03	0.64 ± 0.12	0.61 ± 0.15	0.7971	0.4272	0.54 ± 0.01	0.51 ± 0.09	1.4707	0.1444	0.68 ± 0.12	0.65 ± 0.15	1.1885	0.2374
* 9—Dorsolateral prefrontal cortex	04	0.6 ± 0.11	0.6 ± 0.1	−0.0185	0.9853	0.57 ± 0.01	0.55 ± 0.09	1.0231	0.3086	0.66 ± 0.11	0.65 ± 0.1	0.4286	0.6691
* 46—Dorsolateral prefrontal cortex	05	0.53 ± 0.16	0.54 ± 0.14	−0.1626	0.8712	0.48 ± 0.02	0.5 ± 0.11	−0.5690	0.5706	0.57 ± 0.17	0.58 ± 0.15	−0.2154	0.8299
* 10—Frontopolar area	06	0.62 ± 0.07	0.6 ± 0.08	1.0348	0.3032	0.6 ± 001	0.6 ± 0.04	−0.7637	0.4468	0.69 ± 0.06	0.68 ± 0.06	0.7059	0.4818
* 10—Frontopolar area	07	0.62 ± 0.13	0.63 ± 0.16	−0.2504	0.8028	0.53 ± 0.01	0.54 ± 0.11	−0.3812	0.7039	0.67 ± 0.14	0.68 ± 0.16	−0.3188	0.7505
* 46—Dorsolateral prefrontal cortex	08	0.64 ± 0.07	0.56 ± 0.13	3.6304	0.0004 *	0.58 ± 0.01	0.54 ± 0.12	2.0967	0.0384 *	0.7 ± 0.06	0.63 ± 0.15	3.6598	0.0004 *
45—pars triangularis Broca’s area	09	0.45 ± 0.2	0.48 ± 0.17	−0.7149	0.4763	0.41 ± 0.02	0.44 ± 0.16	−0.7777	0.4385	0.48 ± 0.22	0.52 ± 0.19	−0.7838	0.4350
* 10—Frontopolar area	10	0.58 ± 0.1	0.58 ± 0.1	−0.2765	0.7827	0.56 ± 0.01	0.56 ± 0.07	0.2711	0.7869	0.64 ± 0.1	0.64 ± 0.1	−0.2684	0.7889
* 10—Frontopolar area	11	0.61 ± 0.08	0.62 ± 0.05	−0.4760	0.6351	0.6 ± 0.01	0.61 ± 0.02	−1.1720	0.2439	0.69 ± 0.06	0.7 ± 0.03	−0.6768	0.5000
* 10—Frontopolar area	12	0.62 ± 0.08	0.62 ± 0.08	0.0643	0.9489	0.6 ± 0.01	0.59 ± 0.06	0.5009	0.6175	0.69 ± 0.07	0.69 ± 0.08	0.2394	0.8113
* 46—Dorsolateral prefrontal cortex	13	0.58 ± 0.09	0.55 ± 0.12	1.4942	0.1382	0.58 ± 0.01	0.58 ± 0.06	−0.0972	0.9228	0.65 ± 0.08	0.62 ± 0.11	1.5247	0.1304
* 46—Dorsolateral prefrontal cortex	14	0.53 ± 0.13	0.51 ± 0.16	0.4713	0.6384	0.5 ± 0.01	0.48 ± 0.13	0.9824	0.3282	0.58 ± 0.14	0.56 ± 0.17	0.4336	0.6655
* 10—Frontopolar area	15	0.47 ± 0.18	0.51 ± 0.17	−1.0407	0.3004	0.43 ± 0.02	0.48 ± 0.14	−1.4789	0.1422	0.51 ± 0.19	0.56 ± 0.18	−1.1735	0.2433
* 10—Frontopolar area	16	0.61 ± 0.07	0.63 ± 0.09	−1.1105	0.2694	0.6 ± 0.01	0.59 ± 0.05	0.6103	0.5430	0.68 ± 0.05	0.69 ± 0.07	−0.9391	0.3499
* 11—Orbitofrontal area	17	0.44 ± 0.19	0.41 ± 0.2	0.7440	0.4585	0.39 ± 0.02	0.36 ± 0.16	0.9054	0.3673	0.48 ± 0.21	0.43 ± 0.22	0.9364	0.3513
* 46—Dorsolateral prefrontal cortex	18	0.53 ± 0.14	0.51 ± 0.18	0.5957	0.5527	0.5 ± 0.01	0.48 ± 0.14	0.8376	0.4042	0.57 ± 0.15	0.55 ± 0.19	0.6609	0.5101
* 11—Orbitofrontal area	19	0.46 ± 0.16	0.48 ± 0.19	−0.4376	0.6626	0.43 ± 0.02	0.45 ± 0.13	−0.4082	0.6840	0.5 ± 0.18	0.52 ± 0.19	−0.3948	0.6938
* 11—Orbitofrontal area	20	0.52 ± 0.13	0.56 ± 0.13	−1.3365	0.1843	0.52 ± 0.01	0.54 ± 0.1	−0.9959	0.3216	0.58 ± 0.14	0.62 ± 0.13	−1.2797	0.2035
* 11—Orbitofrontal area	21	0.44 ± 0.17	0.48 ± 0.19	−1.0392	0.3011	0.42 ± 0.02	0.43 ± 0.15	−0.3884	0.6985	0.47 ± 0.19	0.51 ± 0.2	−0.9037	0.3682
* 11—Orbitofrontal area	22	0.41 ± 0.18	0.44 ± 0.16	−0.6393	0.5241	0.38 ± 0.02	0.44 ± 0.13	−1.7044	0.0913	0.44 ± 0.2	0.48 ± 0.18	−0.8278	0.4097

Note: ROI, region of interest; CH, channel. T-value and *p*-value were the results of the two-sample test (*p* < 0.05). * indicates that the results passed the two-sample test.

## Data Availability

The data that support the findings of this study are available from the corresponding author upon reasonable request.
